# Individual Variability and Test-Retest Reliability Revealed by Ten Repeated Resting-State Brain Scans over One Month

**DOI:** 10.1371/journal.pone.0144963

**Published:** 2015-12-29

**Authors:** Bing Chen, Ting Xu, Changle Zhou, Luoyu Wang, Ning Yang, Ze Wang, Hao-Ming Dong, Zhi Yang, Yu-Feng Zang, Xi-Nian Zuo, Xu-Chu Weng

**Affiliations:** 1 Fujian Provincial Key Lab of the Brain-like Intelligent systems, Xiamen University School of Information Science and Engineering, Xiamen, Fujian 361005, China; 2 Zhejiang Key Laboratory for Research in Assessment of Cognitive Impairments, Center for Cognition and Brain Disorders, Hangzhou Normal University, Hangzhou, Zhejiang 311121, China; 3 Key Laboratory of Behavioural Sciences and Magnetic Resonance Imaging Research Center, Institute of Psychology, Chinese Academy of Sciences, Beijing 100101, China; 4 University of Chinese Academy of Sciences, Beijing 100049, China; 5 Laboratory for Functional Connectome and Development, Institute of Psychology, Chinese Academy of Sciences, Beijing 100101, China; 6 Faculty of Psychology, Southwest University, Beibei, Chongqing 400715, China; 7 Department of Psychology, School of Education Science, Guangxi Teachers Education University, Nanning, Guangxi 530001, China; Beijing Normal University, CHINA

## Abstract

Individual differences in mind and behavior are believed to reflect the functional variability of the human brain. Due to the lack of a large-scale longitudinal dataset, the full landscape of variability within and between individual functional connectomes is largely unknown. We collected 300 resting-state functional magnetic resonance imaging (rfMRI) datasets from 30 healthy participants who were scanned every three days for one month. With these data, both intra- and inter-individual variability of six common rfMRI metrics, as well as their test-retest reliability, were estimated across multiple spatial scales. Global metrics were more dynamic than local regional metrics. Cognitive components involving working memory, inhibition, attention, language and related neural networks exhibited high intra-individual variability. In contrast, inter-individual variability demonstrated a more complex picture across the multiple scales of metrics. Limbic, default, frontoparietal and visual networks and their related cognitive components were more differentiable than somatomotor and attention networks across the participants. Analyzing both intra- and inter-individual variability revealed a set of high-resolution maps on test-retest reliability of the multi-scale connectomic metrics. These findings represent the first collection of individual differences in multi-scale and multi-metric characterization of the human functional connectomes in-vivo, serving as normal references for the field to guide the use of common functional metrics in rfMRI-based applications.

## Introduction

Functional connectomics with resting-state functional magnetic resonance imaging (rfMRI) is a widely used tool to map the intrinsic architecture in the human brain [1–4]. Different computational methods have been developed to examine the functional changes associated with development and disease processes [5, 6]. The rfMRI-derived metrics can be categorized into three different types according to the spatial scale at which they characterize human brain function, namely the local scale (brain areas/regions), meso-scale (subnetworks or modules), and global brain. At each scale these metrics aim to characterize information segregation and integration in functional connectomics. Functional connectivity between brain areas can be defined in a relatively straightforward manner with temporal correlations [7, 8]; other measures of connectivity can be used, but may be difficult hard to interpret, partly because there are many confounds [9] that can affect these metrics [10–13]. Fortunately, several recent studies have made efforts on explore the potential neurobiological significance of these metrics [14–16].

High test-retest reliability is a necessary requirement for developing a biomarker of functional connectomics for clinical application [17]. The existing rfMRI-derived metrics have demonstrated moderate to high test-retest reliabilities and are widely used to study different aspects of the human brain’s intrinsic functional architecture or functional connectomics [18], and systematically reviewed in [19]. Specifically, at local scales, the amplitude measures exhibit moderate to substantial reliability [20] whereas the local functional homogeneity metrics are highly reliable (substantial to almost perfect) [21]. At the scale of networks, common intrinsic connectivity networks show relatively high reliability while the seed-based methods [22] are relatively less reliable than independent component analysis [23]. At scales of the entire connectome, various centrality metrics exhibit only fair to moderate test-retest reliability [2], although this can be affected by sampling rates, preprocessing and computational strategies [24–27]. Test-retest reliability of these metrics have been partly examined in typically [28, 29] or atypically developing children [29] as well as normal [30–32] and abnormal aging people [33].

Test-retest reliability is an integrative statistical measure of both intra-individual variability (intraVar) and inter-individual variability (interVar) [34, 35], composing so-called individual differences. Previous studies have presented significant individual differences in human brain intrinsic function at both the group and individual level [1, 15, 36–38], although most focused on seed-based functional connectivity. The distribution of individual differences to within- and between-subject variability determines the degree of test-retest reliability of the functional measures. However, very few studies have explored details of both intraVar and interVar of common functional measurements due to lacking a richly-sampled datasets within and between individuals. One representative work on interVar was recently conducted by Mueller and colleagues [39], shedding light on the role of intrinsic functional connectivity in brain evolution and development by relating individual differences to interVar of cortical morphology, anatomical distance and cognition. This dataset has been employed as normal reference in guiding individual-level network parcellation of the human cortex [40]. Another seminal work investigated the spatial structure of intraVar of whole-brain intrinsic connectivity within one-hour rfMRI scans from 10 participants and concluded that this intraVar also reflected functionally specialized and flexible configuration across the human brain cerebral cortex [41]. A recent work on the MyConnectome project [42] by Poldrack and colleagues has yielded the most detailed depiction of an individual brain’s function across one year (almost daily scanned) [43], together with the work from Choe and colleagues on rfMRI-derived networks of an individual across a period of 3.5 years (weekly scanned) [44], further highlighting the value of mapping intra-individual variability more accurately to probe the intrinsic function.

While the mapping of the individual differences are fundamentally important to understand how the human brain organizes, changes dynamically, as well as how it is altered by disease conditions [45, 46], a comprehensive study of both intraVar and interVar of the rfMRI measures in a single sample is still missing. The major challenge is to obtain a longitudinal dataset with enough samples at both the group and individual levels. In the present research, we scanned 30 healthy adults using rfMRI with a one-month longitudinal experiment design. This allowed each participant to undergo an rfMRI scanning session every 2–3 days, resulting in ten repeated measurements for each participant. This test-retest sample has been released (http://dx.doi.org/10.15387/fcp_indi.corr.hnu1) as part of the Consortium for Reliability and Reproducibility (CoRR) [47], which shares the data via the Neuroimaging Informatics Tools and Resources Clearinghouse (NITRC). It is also available at FigShare (https://figshare.com/s/7dac285e153e176d90e8) with a digital object identifier (10.6084/m9.figshare.2007483). Using this dataset, we 1) implement surface-based computation of the common rfMRI metrics at scales of local areas, subnetwork/module and global connectome; 2) delineate both intraVar and interVar of the common rfMRI metrics at the three different spatial scales; 3) examine test-retest reliability of these common brain connectomics metrics and generate their cortical surfaces; 4) related these individual differences and the test-retest reliability to the brain networks and the cognitive components in the intrinsic functional architecture.

## Methods and Analysis

### Participants

Thirty participants aged 20 to 30 years old (15 females, mean age = 24, SD = 2.41) were recruited and scanned ten times over approximately one month. None of participants had a history of neurological or psychiatric disorders, substance abuse, or head injury with loss of consciousness. The ethics committee of the Center for Cognition and Brain Disorders (CCBD) at Hangzhou Normal University approved this study. Written informed consent was obtained from each participant prior to data collection.

### MRI Data Acquisition

MRI Imaging sessions were performed using a GE MR750 3.0 Tesla scanner (GE Medical Systems, Waukesha, WI) at CCBD, Hangzhou Normal University. Each participant underwent ten imaging scans over one month with one scan every three days, during which two imaging sequences were completed to measure individual brain structure and function. Specifically, a T2-weighted echo-planar imaging (EPI: TR = 2000 ms, TE = 30 ms, flip angle = 90°, field of view = 220 × 220 mm, matrix = 64 × 64, voxel size = 3.4 × 3.4 × 3.4 mm, 43 slices) sequence was performed to obtain resting state fMRI images for 10 minutes. A T1-weighted Fast Spoiled Gradient echo (FSPGR: TR = 8.1 ms, TE = 3.1 ms, TI = 450 ms, flip angle = 8°, field of view = 256 × 256 mm, matrix = 256 × 256, voxel size = 1.0 × 1.0 × 1.0 mm, 176 sagittal slices) was carried out to acquire a high-resolution anatomical image of the brain structure. To minimize head movement, straps and foam pads were used to fix the head snugly during each scan. The participants were instructed to relax and remain still with their eyes open, not to fall asleep, and not to think about anything in particular. The screen presented a black fixation point ‘+’ in the center of the gray background. After the scans, all the participants were interviewed, and none of them reported to have fallen asleep in the scanner. The time of day of MRI acquisition was controlled within participants.

### MRI Data Preprocessing

The Connectome Computation System (CCS: https://github.com/zuoxinian/CCS) was developed to provide a multimodal image analysis platform for the discovery science of human brain function by integrating three main MRI data processing packages [48–50] with our MATLAB implementations of various computational modules for image quality control, surface-based rfMRI metrics, data mining algorithms, reliability and reproducibility assessments and visualization. The primary CCS pipeline consists of both anatomical and functional processing, which are documented in the CCS paper in details [51]. Here, we only provide an overall description of these steps.

The CCS anatomical pipeline first removed noise from individual MR T1 images by adopting a spatially adaptive non-local means filter [52, 53]. For each participant, regarding that the focus of the present analysis is changes of rfMRI metrics, we aligned and averaged the ten de-noised T1 images to produce a robust, high-resolution anatomical image with high contrast between gray matter (GM) and white matter (WM) for generation of an individual cortical surface model as anatomical reference. Using this image, the skull was then stripped and manually edited for a better brain extraction, and the 3D extracted brain volume was segmented into different tissues such as cerebrospinal fluid (CSF), WM, GM and further parcellation of the GM tissue. With such information, individual pial (GM/CSF boundary) and white (GM/WM boundary) surfaces were reconstructed and spatially normalized to match a group-level standard template surface in the Montreal Neurological Institute (MNI) space via a sphere registration [49, 54, 55].

The subsequent CCS functional pipeline discarded the first 5 EPI volumes (10 seconds), removed and interpolated temporal spikes (see instructions in [56, 57] regarding in-scanner head motion), corrected acquisition timing among image slices and head motion among image volumes and normalized the 4D global mean intensity to 10,000. White surfaces were then employed by a boundary-based registration (BBR) algorithm to match spatial correspondences between individual functional images to anatomical images [58]. To further eliminate the effect of head motion and physiological noises during the rfMRI scanning session, we regressed out the estimated Friston’s 24-parameter motion curves [37] and nuisance signals measured as WM and CSF mean time series [59] from individual rfMRI time series. Linear and quadratic trends were also regressed out from the rfMRI data by multiple linear regressions. Finally, the data were projected onto the *fsaverage* surface grid (average inter-vertex distance = 1 mm) and down-sampled to the *fsaverge5* surface grid (average inter-vertex distance = 4 mm) for subsequent rfMRI computation.

### Participant-level rfMRI Metrics Derivation

**Fig 1:**
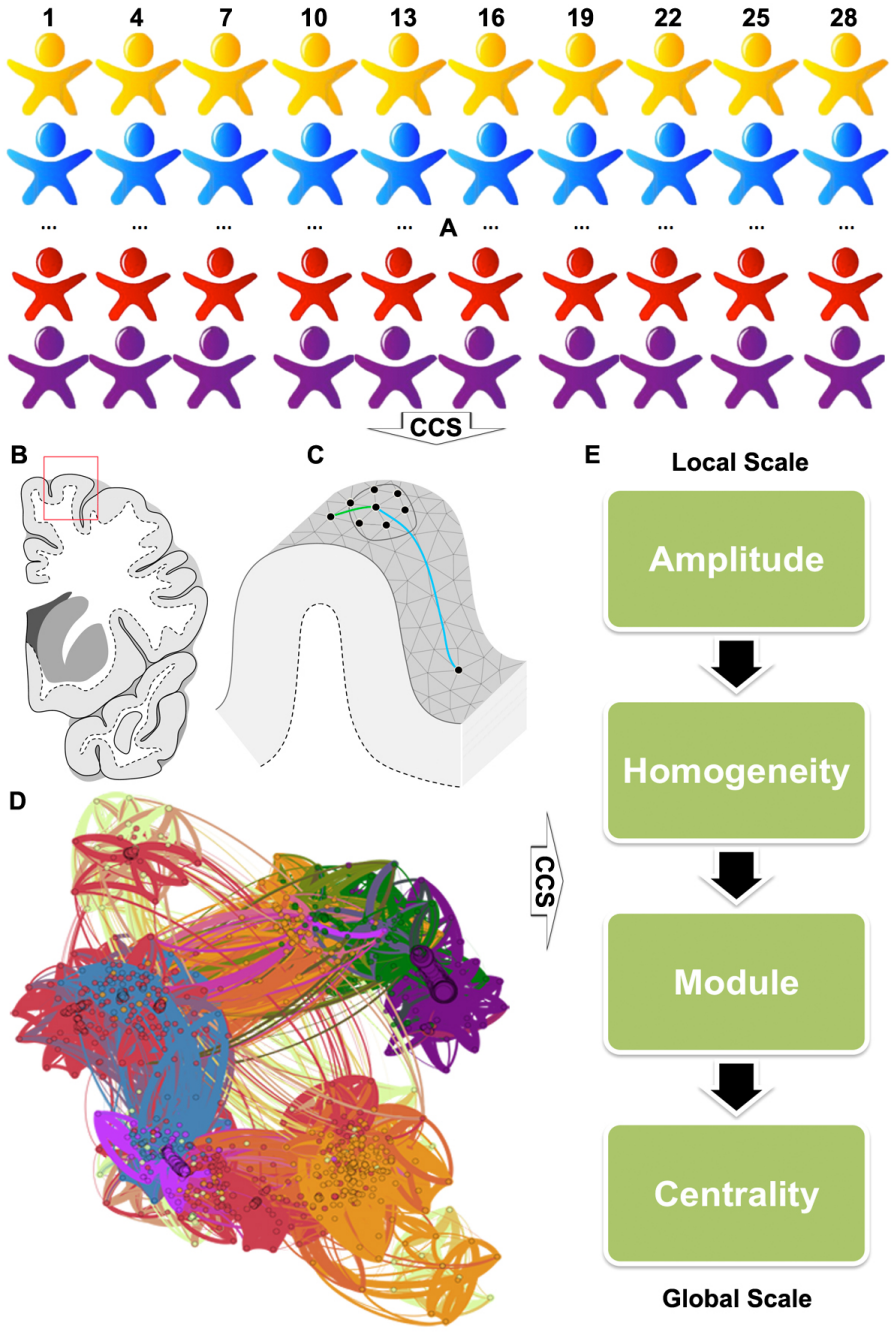
The overall analytic strategy implemented in the Connectome Computation System (CCS). All individual rfMRI images (A) are first preprocessed through the CCS pipeline and then CCS projected individual rfMRI time series onto a uniform cortical surface grid based upon the brain tissue classification (B/C). As a proof of concept, an individual-level functional connectome can be modelled as a graph or network with vertices as nodes and pair-nodes dependency as edges (D). To achieve a systematic and comprehensive characterization of the brain graph, we proposed a set of metrics at three different scales from a single vertex to the whole cortex for measuring the amplitude and homogeneity at local scales, subnetworks or modules at meso-scales, and connectome centrality at the global scale (E). The connectome graph is reproduced and modified from [15].

The overall analytic strategy is presented in Fig 1. The above CCS pipeline preprocessed all individual rfMRI images (Fig 1A) and projected individual rfMRI time series onto a uniform cortical surface grid (*fsaverge5*) based upon the brain tissue classification (Fig 1B and 1C). As a proof of concept, an individual-level functional connectome can be modeled as a graph or network with vertices as nodes and pair-nodes dependency as edges (Fig 1D). To achieve a systematic and comprehensive characterization of the brain graph, we proposed a set of metrics at three different scales from a single vertex to the whole cortex (Fig 1E). Given an arbitrary vertex *v*_*i*_(*i* = 1, ⋯, *V*) on the surface where *V* is the number of all vertices of the *fsaverge5* surface grid, its time series measured with rfMRI is *v*_*i*_(*t*_*j*_)(*j* = 1, ⋯, *T*) where *T* is the number of time points of the rfMRI scan (assume an even number). All these metrics are summarized in this section with computational details.

#### Scales of Local Area

To measure local-scale characteristics of the human brain function, two amplitude metrics were employed, namely, the amplitude of low frequency fluctuation (ALFF) [10] and its fractional version (fALFF) [11]. Both ALFF and fALFF were derived from spatially smoothed rfMRI data (6 mm full-width at half-maximum isotropic Gaussian kernel) via the Fourier decomposition [20]. ALFF measures the strength or intensity of the low-frequency Ω = [0.01,0.1] (Hz) oscillations whereas fALFF is the relative amplitude contribution of the specific frequency to the whole detectable frequency range Ω_0_ = (0,1/(2Δ*t*)] (Hz) where Δ*t* is the sampling rate Eq (1).

$$\begin{array}{ccc} v_{i}\left(t\right) & = & \sum_{l=1}^{T/2}\{\alpha_{l}\cos\left(\omega_{l}t\right)+\beta_{l}\sin\left(\omega_{l}t\right)\},\omega_{l}=2\pi l/T\\ \text{ALFF}(v_{i},\Omega ) & = & \sqrt{\sum_{l:\omega_{l}\in \Omega }\frac{\alpha {\left(\omega_{l}\right)}_{l}^{2}+\beta {\left(\omega_{l}\right)}_{l}^{2}}{T/2}},\;\text{fALFF}\left(v_{i}\right)=\frac{\text{ALFF}(v_{i},\Omega )}{\text{ALFF}(v_{i},\Omega_{0})} \end{array}$$ (1)

With a relatively larger scale than the two amplitude measures, to measure local connectivity, regional homogeneity (ReHo) was also employed to characterise the local functional homogeneity of each vertex across the cortical mantle [12, 14, 15, 21]. This surface-based ReHo was derived from the non-smoothed but temporally band-pass filtered (the passing frequency band Ω) rfMRI data. The Kendall’s coefficient of concordance of time series within a set of neighboring vertices quantifies the ReHo. Denote $\gamma_{i}^{j}$ as the rank time series of *v*_*i*_(*t*_*j*_), and the Eq (2) formulates ReHo computation where K is the number of neighbors of the vertex *v*_*i*_, *N*(*v*_*i*_). The mean rank across its neighbors at the *j*-th time point is ${\bar{\gamma }}_{i}^{j}$, and its overall mean rank across all neighboring voxels and time points is ${\bar{\gamma }}_{i}$. Here, two neighbor-sizes used for computation of homogeneity metrics, namely ReHo1 (K = 7) and ReHo2 (K = 20).

$$\begin{array}{c} \text{ReHo}\left(v_{i},N\left(v_{i}\right)\right)=\frac{\sum_{j=1}^{T}{\left(\gamma_{i}^{j}\right)}^{2}-T{\left({\bar{\gamma }}_{i}\right)}^{2}}{\frac{1}{12}K^{2}(T^{3}-T)}=12\frac{\sum_{j=1}^{T}{\left({\bar{\gamma }}_{i}^{j}\right)}^{2}}{\left(T^{3}-T\right)}-3\frac{T+1}{T-1}. \end{array}$$ (2)

#### Scales of Subnetwork

Seed-based method can construct a subnetwork of the seed region. Denote the representative time series of the seed as a vector $s={\left(s^{j}\right)}_{j=1}^{T}$, and the full brain rfMRI time series as a matrix $V=\left(v_{i}^{j}\right)={\left(v_{i}\right)}_{i=1}^{V}$. Pearson’s correlation coefficient between **s** and **v**_*i*_ is defined as *ρ* in the Eq (3) and further converted into Fisher-z value to quantify the seed-based functional connectivity (SFC) between the seed and the vertex. This method can be sensitive to the seed selection, and here we choose a small default network region from a highly reproducible functional parcellation of the human brain [60], namely ‘17Networks_LH_DefaultA_PCC’. Its representative time series was obtained by averaging all time series within the seed area. Of note, this surface-based SFC was estimated using the same preprocessed rfMRI data as ReHo but normalized (0 mean and 1 variance).

$$\begin{array}{c} \text{SFC}\left(v_{i},s\right)=\frac{1}{2}\ln(\frac{1+\rho (v_{i},s)}{1-\rho (v_{i},s)})\;\;\text{where}\;\;\rho \left(v_{i},s\right)=sv_{i}^{{}^{\prime }}. \end{array}$$ (3)

A fast MATLAB implementation can be achieved for the entire cortex correlation with the seed region as in the Eq (4) [2, 51].

$$\begin{array}{c} \text{SFC}\left(V,s\right)=Vs^{\prime }=(v_{1}s^{\prime },\cdots ,v_{V}s^{\prime }) \end{array}$$ (4)

To extract multiple networks simultaneously from individual rfMRI time series (not normalized), a dual regression (DR) procedure was employed [23]. Specifically, spatial confidence maps $y_{i=1,\cdots ,7}^{\left(1\right)}$ of the seven networks derived from 1,000 healthy adults [60] of the Brain Genomics Superstruct Project [61] were employed in the first regression on individual rfMRI images to construct the characteristic time series $x_{i=1,\cdots ,7}^{\left(1\right)}$ of the seven networks at individual level in Eq (5). These characteristic time series were further entered into the second regression on the individual rfMRI time series to extract individual spatial maps of the seven networks $y_{i=1,\cdots ,7}^{\left(2\right)}$ in Eq (6).

$$\begin{array}{c} {\text{DR}}^{\left(1\right)}(V,y_{i=1,\cdots ,7}^{\left(1\right)}):\;V^{\prime }=(y_{1}^{\left(1\right)},\cdots ,y_{7}^{\left(1\right)}){\left(x_{1}^{\left(1\right)},\cdots ,x_{7}^{\left(1\right)}\right)}^{\prime }+E^{\left(1\right)} \end{array}$$ (5)

$$\begin{array}{c} {\text{DR}}^{\left(2\right)}(V,x_{i=1,\cdots ,7}^{\left(1\right)}):\;V=(x_{1}^{\left(1\right)},\cdots ,x_{7}^{\left(1\right)}){\left(y_{1}^{\left(2\right)},\cdots ,y_{7}^{\left(2\right)}\right)}^{\prime }+E^{\left(2\right)} \end{array}$$ (6)

#### Scales of Global Connectome

At this scale, an individual functional connectome was reconstructed by computing the full cortical correlation matrix **V** **V**′. This surface-based computation constructed weighted graphs for individual brains by quantifying the inter-vertex connection as the Pearson’s correlation between their preprocessed rfMRI signals (not smoothed but temporally band-pass filtered) [2]. All these cortical graphs were set to have the same edge density (0.05) to make them comparable across participants and time. Denote the adjacency matrix as in Eq (7).

$$\begin{array}{c} A=(a_{ij})=\frac{1}{2}\ln(\frac{1+\rho (v_{i},v_{j})}{1-\rho (v_{i},v_{j})}),i,j=1,\cdots ,V \end{array}$$ (7)

We applied degree centrality (DCw)[2, 62] and eigenvector centrality (ECw) [2, 63, 64] to capture the feature of the information processing in the weighted functional connectomes. DCw is the degree of the functional connectivity by measuring the sum of weighted connections for each node vertex Eq (8). ECw is the eigenvector corresponding to the maximal eigenvalue *λ*_1_ of the adjacency matrix Eq (8) and captures an aspect of centrality that extends to global features of the weighted brain graph.

$$\begin{array}{c} {\text{DC}}_{w}\left(v_{i}\right)=\sum_{j=1}^{V}a_{ij},\;\;{\text{EC}}_{w}\left(v_{i}\right)=\frac{1}{\lambda_{1}}\sum_{j=1}^{V}a_{ij}{\text{EC}}_{w}\left(v_{j}\right) \end{array}$$ (8)

### Group-level Statistical Assessments

To examine the dynamic changes of human brain function for individual subjects and among different subjects over one month, we applied the Linear Mixed Effects (LME) models to 300 samples of each functional metric. We also included various potential confounding factors in LMMs such as the age, sex, mean frame-wise displacement (meanFD) of head motion, and BBR minimum cost (mcBBR) at the participant-level.

At the whole-brain level, for each specific functional metric M ∈ {ALFF, fALFF, ReHo1, ReHo2, SFC, DR, DCw, ECw}, we employed the model as in Eq (9) to estimate both intraVar and interVar. We denote gM_*ij*_ as the global mean metric of the *i*-th measurement of the *j*-th participant (for *i* = 1⋯10 and *j* = 1⋯30).

$$\begin{array}{c} {\text{gM}}_{ij}=\mu_{00}+\gamma_{0j}+{\text{age}}_{j}+{\text{sex}}_{j}+{\text{motion}}_{ij}+\epsilon_{ij} \end{array}$$ (9)

Interplays between each pair of the DR-derived networks were estimated as the temporal correlation between each pair of their characteristic time series. This interplay correlational matrix was converted to Fisher’s z-values. An LME model Eq (9) was then applied to each of the interplay metric for estimation of their variance components.

At the vertex-level, Eq (9) was further refined by including the global mean metric as a covariate to assess the network-specific variability of the metric within and between subjects. Such a strategy of post-hoc standardization in functional connectomics has been proposed recently and is more efficient and reliable to detect individual differences [38]. Specifically, the regression model, as shown in Eq (10), was applied to each vertex on the *fsaverage5* surface and yielded vertex-wise statistical assessments. To account for heterogeneous changes in regional volume, the amount of regional volume change required to warp a subject into the standard surface, *fsaverage*, is measured using the vertex-wise covariate derived from the Jacobian determinant of the spherical transform in FreeSurfer.

$$\begin{array}{ccc} M_{ij}\left(v\right) & = & \mu_{00}\left(v\right)+{\text{gM}}_{ij}+\gamma_{0j}\left(v\right)+{\text{age}}_{j}+{\text{sex}}_{j}\\ & & +{\text{motion}}_{ij}+{\text{mcBBR}}_{ij}+{\text{Jacobian}}_{j}\left(v\right)+\epsilon_{ij}\left(v\right) \end{array}$$ (10)

LME models (9) and (10) contain parameters of both fixed and random effects, and the random error term. The basic assumption of an LME is that the observed variable and error term are normally distributed with mean 0 and variances. Here, the variance $\sigma_{b}^{2}$ is the inter-individual or between-subject variance (interVar); in other words, the variation between participants while $\sigma_{w}^{2}$ is the intra-individual or within-subject variance representing the variation within single subjects across one month (intraVar). In order to evaluate the test-retest reliability of the functional metrics, the intra-class correlation (ICC) was calculated by according to its definition as $\text{ICC}=\sigma_{w}^{2}/\left(\sigma_{b}^{2}+\sigma_{w}^{2}\right)$. All the estimation of the variances was implemented by CCS. To avoid negative estimation of the ICC, the variance components in the LMMs were estimated with the restricted maximum likelihood (ReML) approach with the covariance structure of compound symmetrical matrix. From this reliability definition, it is clear that the test-retest reliability integrates both intra-individual and inter-individual variability. Low intra-individual variability or high inter-individual variability will lead to high test-retest reliability. The reliability bounds the validity of various metrics used in clinical diagnoses and thus becomes an essential requirement on developing a biomarker in applications [17].

## Results

**Fig 2:**
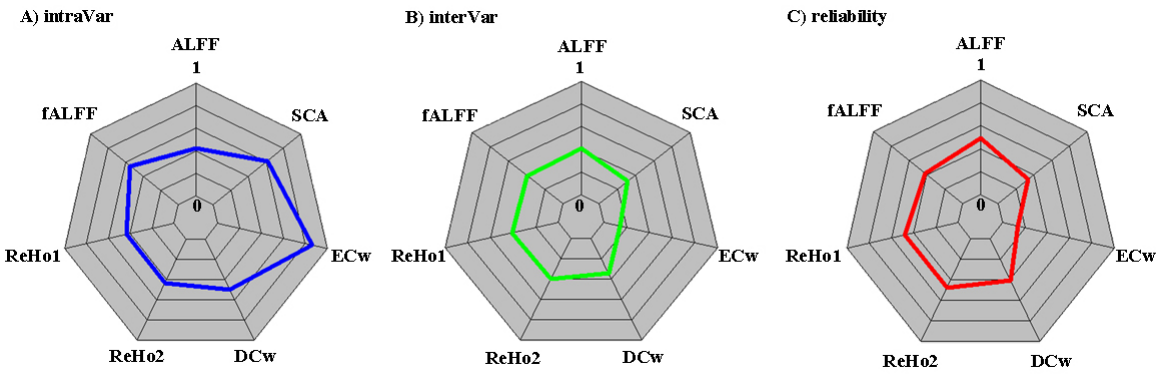
Quantification of variability of seven common rfMRI metrics across the whole cortical mantle. A) Auantification measures of intra-individual variability strength are plotted in polar form for amplitude of low-frequency fluctuations (ALFF), fractional ALFF (fALFF), regional homogeneity with length-one (ReHo1) and length-two (ReHo2) neighbors, seed-based connectivity analysis (SCA), weighted degree centrality (DCw) and eigenvector centrality (ECw). B) Auantification measures of inter-individual variability strength are plotted in polar form for the rfMRI metrics. C) Auantification measures of test-retest reliability strength are plotted in polar form for the rfMRI metrics. Note that for the purpose of visualization, all the strengths are normalized into a standard value between 0 and 1 by dividing the specific variance with the overall variance.

Our analyses produced a set of maps of the individual differences in intrinsic functional architecture by delineating intra-individual, inter-individual variability and test-retest reliability across multiple metrics and multiple spatial scales of the human connectome. Specifically, we computed eight different functional metrics to characterize the human brain connectome at local, meso and global scales: ALFF, fALFF, ReHo1, ReHo2, SFC, DR, DCw and ECw to capture the feature of the information processing in the weighted functional connectomes [2]. Vertex-wise maps of the individual variability and test-retest reliability depicted high-resolution distributions of variability and stability of these functional connectomics. In reporting these findings, we categorized the ICC into five common intervals [65]: 0 < ICC ≤ 0.2 (slight); 0.2 < ICC ≤ 0.4 (fair); 0.4 < ICC ≤ 0.6 (moderate); 0.6 < ICC ≤ 0.8 (substantial); and 0.8 < ICC ≤ 1.0 (almost perfect). Global mean measures of all these metrics (for DR, no global mean calculated regarding its methodological consideration) only exhibited fair to moderate test-retest reliability (Fig 2C), indicating their dynamic nature within subjects (Fig 2A) or limited between-subject variability (Fig 2B).

**Fig 3:**
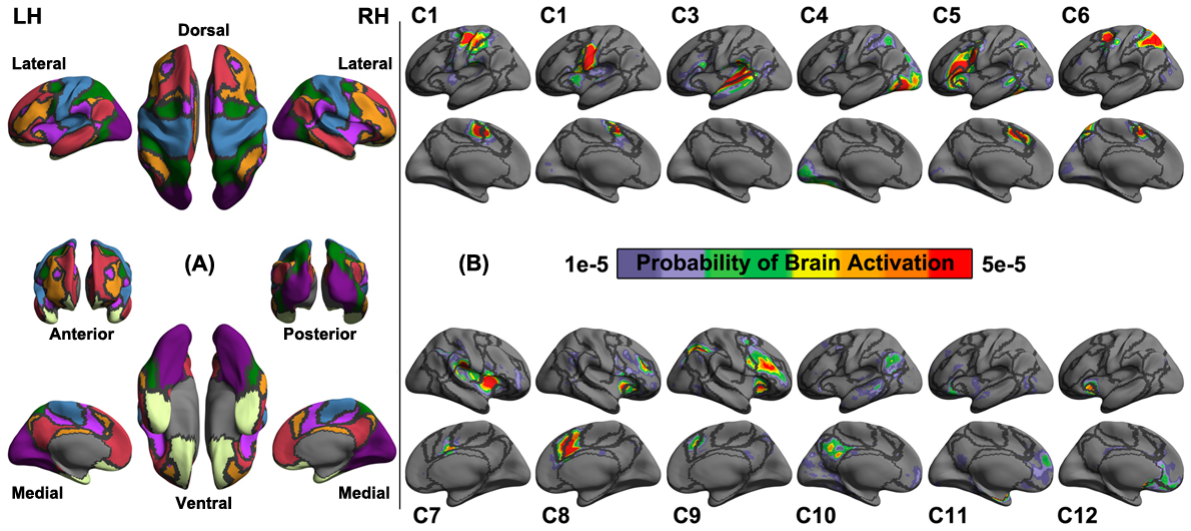
Renders of canonical large-scale networks and cognitive components. A) The seven large-scale networks derived from a large sample (N = 1,000) of healthy resting-state brains [60] including Visual (Purple), Somatomotor (Blue), Dorsal Attention (Green), Ventral Attention (Violet), Limbic (Cream), Frontoparietal Control (Orange), Default (Red). This render projects these networks onto the *fsaverage* surface grid with its dorsal, ventral, lateral, medial, anterior and posterior views of the left hemisphere (LH) and the right hemisphere (RH). Dark gray curves indicate the boundaries between the seven networks. B) A surface render of the cognitive ontology of the brain derived from a large data set of neuroimaging experiments (N = 10,449) that contains twelve (C1-Hand, C2-Mouth, C3-Auditory, C4-Visual, C5-Language, C6-Attention, C7-Autonomic, C8-Inhibition, C9-Working Memory, C10-Default, C11-Basal and C12-Reward) components of cognition [66]. This image is reproduced and modified from [66].

To summarize these findings at multiple scales, together with presentation of these vertex-wise maps, we documented our results in following subsections for the individual differences according their distributions across the common brain networks and cognitive components. The seven large-scale networks derived from 1,000 healthy resting-state brains [60] are rendered in Fig 3: visual (Visual), somatomotor (SomMot), dorsal attention (DorsAttn), ventral attention (VentAttn), limbic (Limbic), frontoparietal control (Control), default (Default) network, and a cognitive ontology of the brain derived from a large data set of neuroimaging experiments (N = 10,449) that contains twelve (C1-Hand, C2-Mouth, C3-Auditory, C4-Visual, C5-Language, C6-Attention, C7-Autonomic, C8-Inhibition, C9-Working Memory, C10-Default, C11-Basal and C12-Reward) components of cognition [66]. Percentages of high values (> = 0.4) and mean values are computed for all the networks and components. This strategy helps to shape the following reports of these large amount findings into a hierarchically organized framework.

### Local Scales: Amplitude and Homogeneity

**Fig 4:**
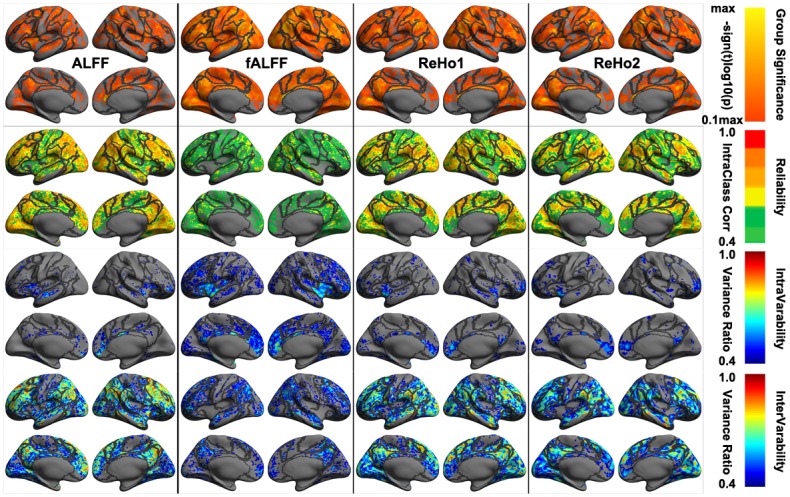
Vertex-wise statistical maps of four common rfMRI metrics at local scales. Maps of group-level statistical significance strength (the first row), test-retest reliability (the second row), intra-individual variability (the third row) and inter-individual variability (the forth row) are rendered onto the *fsaverage* surface grid with its lateral and medial views for amplitude and homogeneity metrics including amplitude of low-frequency fluctuations (ALFF, the first column), fractional ALFF (fALFF, the second column), regional homogeneity with length-one neighbors (ReHo1, the third column) and regional homogeneity with length-two neighbors (ReHo2, the forth column). Dark gray curves indicate the boundaries between the seven canonical neural networks.

These four local metrics demonstrated moderate to almost perfect reliability across the cortex (Fig 4). ALFF and ReHo1, ReHo2 were very similar regarding their spatial patterns of individual differences and reliability whereas fALFF was pretty different from them, showing largely reduced reliability and inter-individual variability. Specifically, regions with the highest reliabilities were primarily located in Visual, DorsAttn, Control and Default networks where intra-individual variability were small, and inter-individual variability were high (S1 Table). An interesting observation is, across all the local metrics, the medial prefrontal cortex node of the default network were the most temporally dynamic region, especially for fALFF. This metric represents the most dynamic one among the four local measures with a focal site in the insular cortex. These highly reliable regions were linked to multiple cognitive components including working memory, attention, default, language, inhibition and visual processes (S2 Table). These high-level cognition processes exhibited low intra-individual variability and high inter-individual variability regarding their local functional characteristics.

### Meso Scales: Networks and Modules

**Fig 5:**
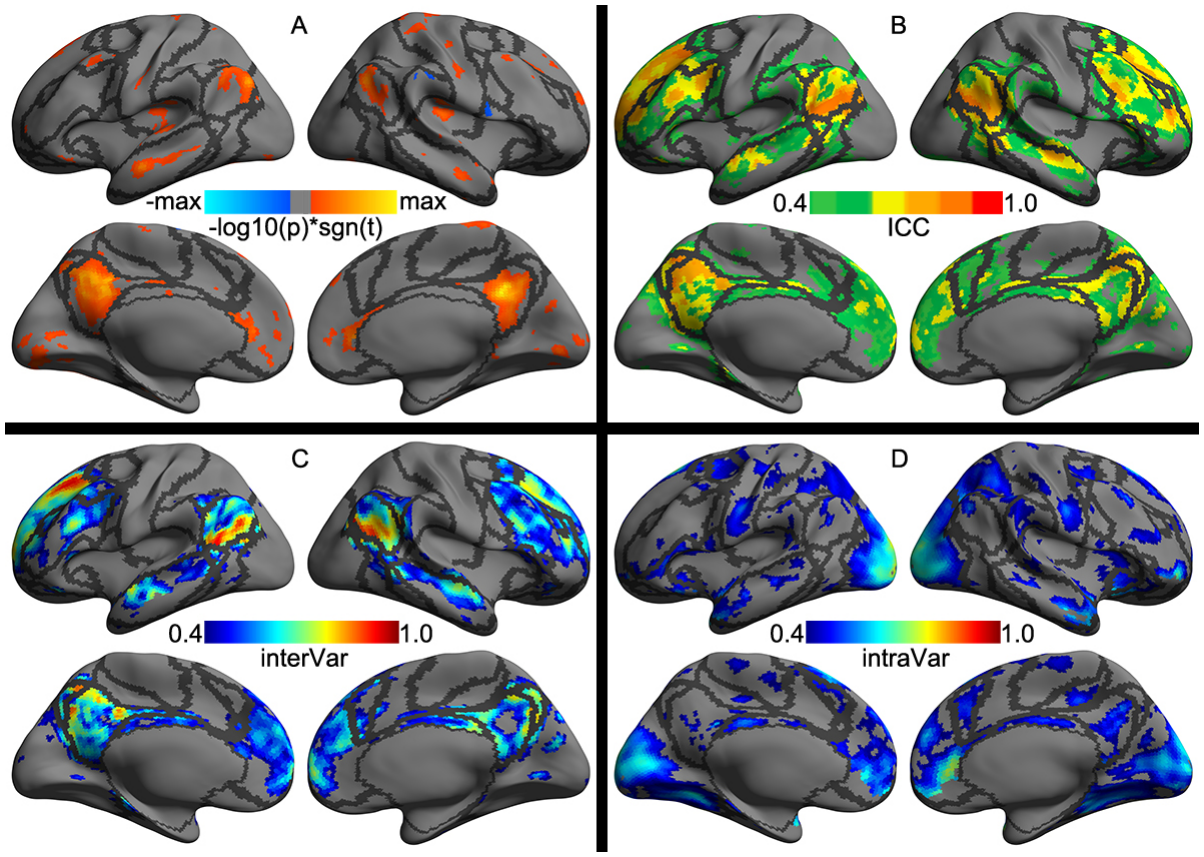
Vertex-wise statistical maps of the seed-based rfMRI connectivity. Maps of group-level statistical significance strength (A), test-retest reliability (B), inter-individual variability (C) and inter-individual variability (D) are rendered onto the *fsaverage* surface grid with its lateral and medial views. Dark gray curves indicate the boundaries between the seven canonical neural networks.

The left PCC region showed substantial test-retest reliability of its seed-based functional connectivity (Fig 5A) with a set of brain areas from both the Default network and Control network (Fig 5B). These two networks covered more than 60% of the reliable PCC’s SFC (S3 Table). Regarding the intra-individual variability, most dynamic SFC of the left PCC mainly connected with the Limbic and the Visual network (Fig 5D). Most test-retest reliable SFC of the PCC were associated with Default, Inhibition, Working Memory and Basal components of the human cognition, indicating low-level within-subject and high-level between-subject variability (S4 Table).

**Fig 6:**
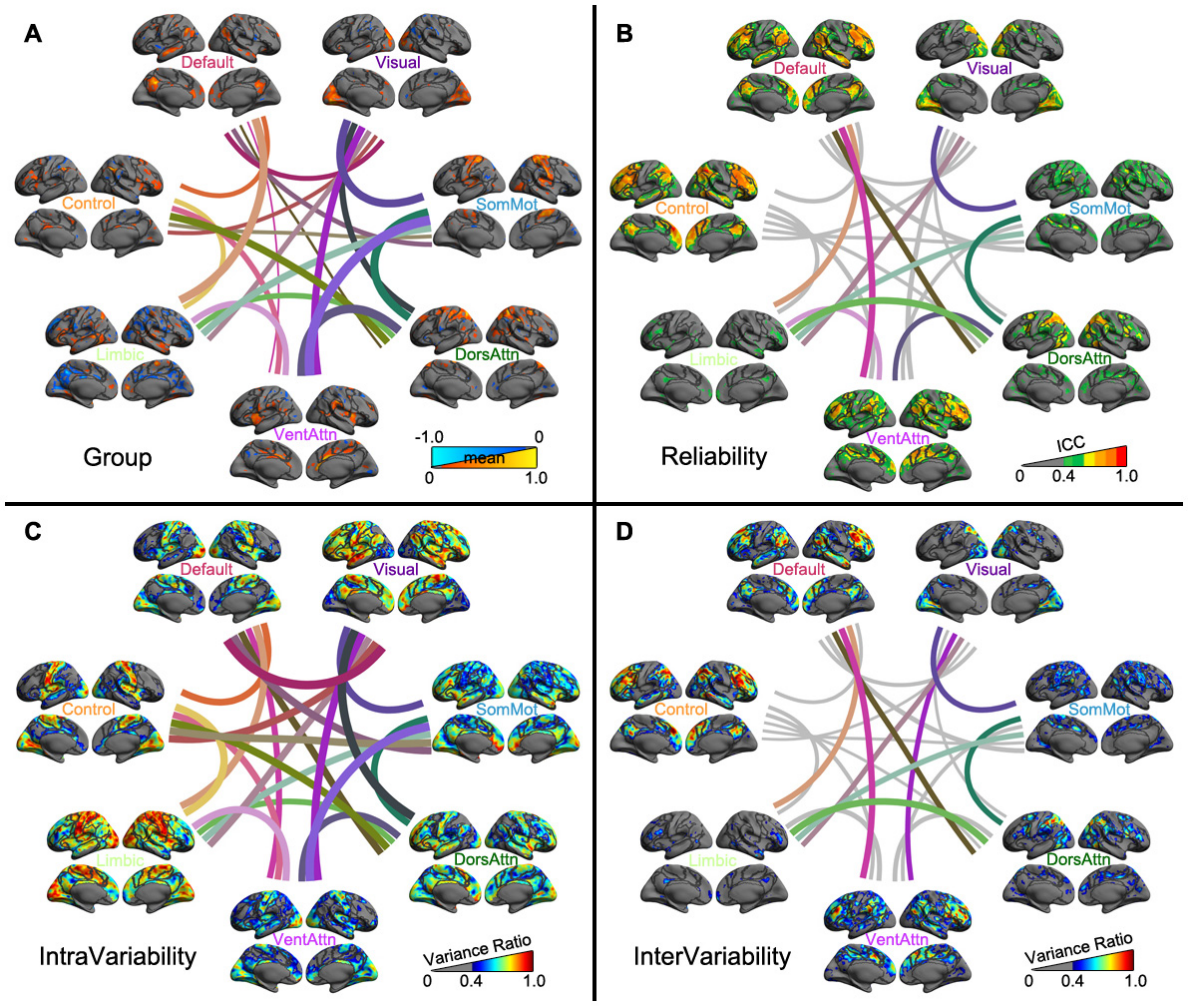
Vertex-wise statistical maps of large-scale common modular metrics at meso scales of subnetworks. Maps of group-level significance strength (A), test-retest reliability (B), intra-individual variability (C) and inter-individual variability (D) are rendered onto the *fsaverage* surface grid with its lateral and medial views for within-network functional specialization (spatial patterns) and between-network functional integration (temporal interactions or interplays) of the seven common large-scale neural networks: Visual, SomMot, DorsAttn, VentAttn, Limbic, Control, Default. Dark gray curves indicate the boundaries between the seven canonical neural networks. Each connection line is plotted with mixed colors of the two networks it connects.

Spatial patterns of the seven cortical functional modules and their temporal interplays as well as their reliability and variability are presented in Fig 6. DR-derived visual network connectivity reliably detectable within the Visual network and DorsAttn network while its interplays with the SomMot network and the Limbic network were reliable (S3 Table). These functional connectivity spatially distributed to Visual and Attention cognitive components (S4 Table). The reliable SomMot network connectivity existed within itself and the two attention (DorsAttn and VentAttn) networks, and were involved in Hand and Mouth cognitive process. Beyond with the Visual network, its interplays with both the DorsAttn and the Limbic networks were reliable. DR-derived connectivity of the Limbic network were not reliable in general while it had reliable interplays with other networks including Visual, SomMot, DorsAttn, VentAttn and Default networks. No cognitive component contained test-retest reliable DR-derived Limbic connectivity.

DR-derived connectivity of the DorsAttn network were reliably presented with itself and the Control network while its temporal interactions with the VentAttn, Default, SomMot and Limbic networks were reliable. Regarding the related cognition processes, these DR-derived connectivity profiles were linked to Attention, Working Memory and Visual components (S4 Table). The within-network DR connectivity of the VentAttn network and its connectivity with Control, DorsAttn, Default and SomMot networks were test-retest reliable. Dynamic interactions between the VentAttn network and Default, Limbic and DorsAttn networks were also reliably observed. Interestingly, this salience-related network showed reliable connectivity across all the 12 cognitive components, among which Inhibition, Working Memory and Attention were the highest three. Beyond within-network connectivity, the Control network exhibited highly reliable connectivity with the two attention and default networks while no reliable between-network interplays were observed for such a highly flexible network. Working Memory, Inhibition, Language and Attention components all had reliable connectivity with this network. Finally, the Default network DR connectivity were reliably distributed within Default, Control and Attention networks whereas its interplays with the two attention networks and the Limbic network were also test-retest reliable. This kind of reliable connectivity were reflected in cognitive components of Working Memory, Inhibition, Default, Language, Basal and Auditory.

### Global Scales: Connectome

**Fig 7:**
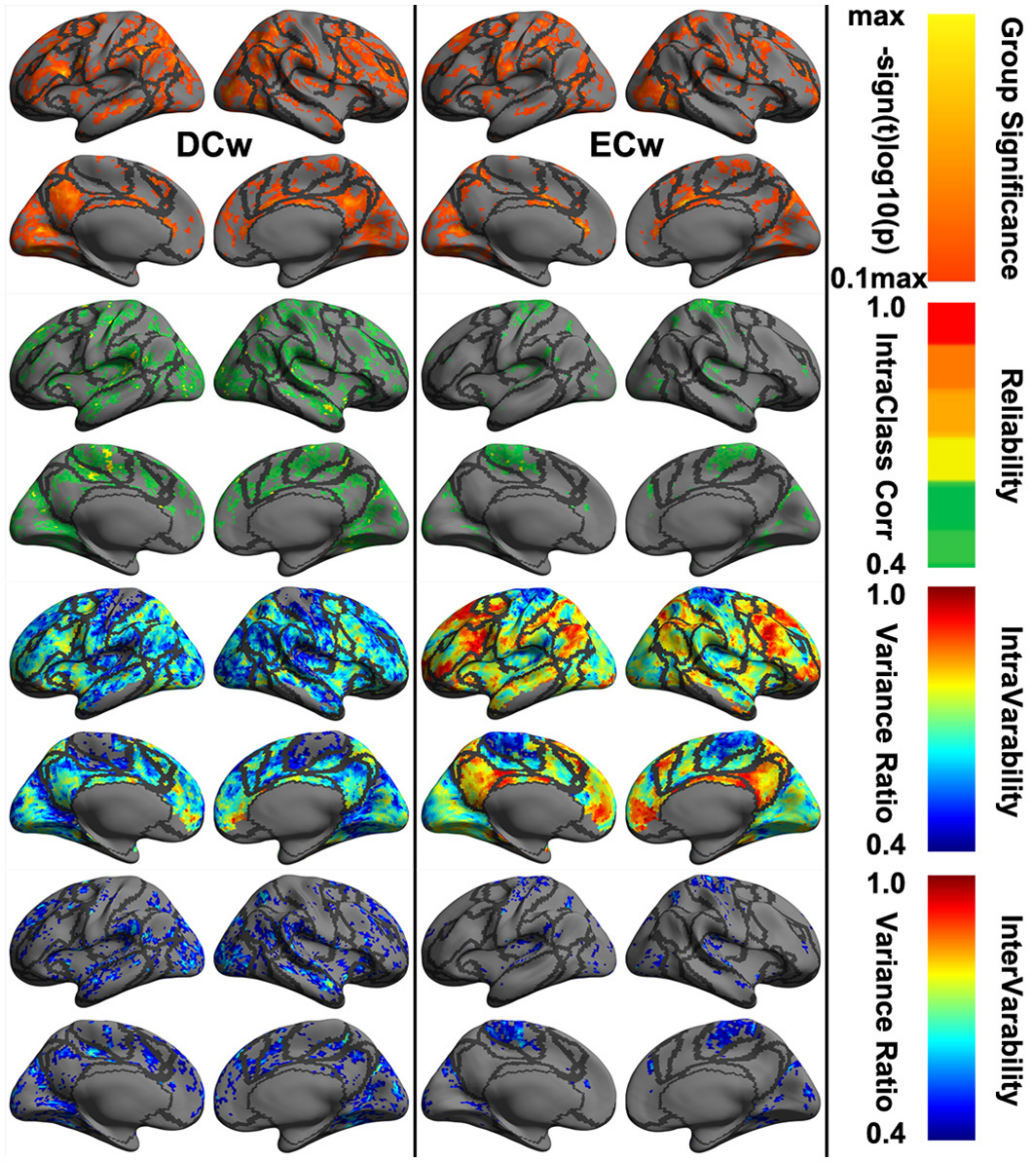
Vertex-wise statistical maps of two common rfMRI metrics at connectome scales. Maps of group-level statistical significance strength (the first row), test-retest reliability (the second row), intra-individual variability (the third row) and inter-individual variability (the forth row) are rendered onto the *fsaverage* surface grid with its lateral and medial views for weighted degree centrality (DCw) and weighted eigenvector centrality (ECw). Dark gray curves indicate the boundaries between the seven canonical neural networks.

As a metric to measure the functionally local connection, DCw characterized the global-scale feature of the human brain connectome. This metric, at vertex-wise level, were only moderately test-retest reliable with very few nodes in parietal and temporal cortex, belonging to Control, Visual and SomMot networks (Fig 7). In contrast, ECw is a functionally global metric and failed in detecting test-retest reliable profiles across the whole cortex although there was a small set of nodes with moderate reliability assignment within parietal areas of the SomMot network (S5 Table). Low test-retest reliability was an indication of the high dynamics across time or intra-individual variability. This high-level instability of the network centrality demonstrated obvious regional specificity, e.g., the highest changes within the Default network. No reliable assignment of the network centrality metrics to the 12 cognitive components was detected (S6 Table).

## Discussion

The functional connectomics field has increasingly appreciated individual differences due to its wide use in both healthy and diseased populations to characterize aspects of brain organization and dynamics. These differences reveal two components, including intra-individual variability (i.e., differences within participants) and inter-individual variability (i.e., differences across participants). An accurate estimation of the two variability sources has been very challenging due to the difficulty of collecting a large neuroimaging sample longitudinally. In the present work, we compute individual differences in common functional connectomics metrics based on large-scale longitudinal neuroimaging data (N = 300) via linear mixed models. For the first time, we delineate a landscape of the individual variability by examining changes of these metrics within and across participants, as well as their test-retest reliability, over a one-month duration. We detected remarkable changes in the variability landscape across different metrics at multiple spatial scales (region, network and connectome). This landscape reveals that more global metrics exhibited larger intra-individual variability across all scales, reflecting the potential inverse relationship between metric complexity and intra-individual variability. In contrast, inter-individual variability demonstrated much more diverse profiles across different metrics, echoing regional differences in functional connectomics at different scales and indicating organizational topology underlying the intrinsic functional architecture in the human brain. Our findings not only depicted this variability landscape but also mapped test-retest reliability of the common functional metrics across one month by integrating both intra-individual and inter-individual variability, providing a resource as references on tests of these variability profiles in brain development and disease conditions.

Resting-state fMRI (rfMRI) represents the most popular tool to investigate functional connectomics currently with a spatial scale of 2–4 millimeters and a temporal scale of 0.5–3 seconds [67, 68]. Temporal dynamics is a hallmark of the human brain functional architecture measured with rfMRI and have been demonstrated as a major contributor to the intra-individual variability across different temporal scales [69–71]. Previous studies have demonstrated that these intra-individual differences could be primarily dominated by the non-stationary components in the rfMRI signals [72]. The time-varying degrees of functional connectivity were commonly thought of as a reflection of flexibility in the functional coordination between different neural systems [45, 73, 74]. Specifically, at the scale of minutes, high intra-individual variability was mainly presented in homotopic functional connectivity as well as non-homotopic between-network connectivity [41]. However, the spatial distribution of the most variable functional connectivity has been a controversy [45, 75, 76]. Our results provide evidence that local features or short-range metrics were more temporally stable than remote features or long-range metrics at scales of days. These parallel to those recent observations at scales of years (development or aging) [77, 78].

Regarding spatial locations, the somatomotor network was the most temporally stable network, and this observation echoed the low intra-individual variability of sensory motor-related cognitive components including hand, mouth and auditory. The limbic network seemed to be most dynamic over the one month, although this might be an indication of the low signal-to-noise ratio during the rfMRI scan within this network. Beyond the two networks, the default network and dorsal attention network were also highly variable over a single month. These findings enriched previous observations of the dynamic functional connectivity at single scales by providing a full picture of its multi-scale spatial distribution (whole brain, networks and voxels). One significant and novel addition from the present work to the existing literature is the one-month temporal stability of 12 components of human cognition. Notably, working memory, inhibition, attention, language and visual process are the five most variable cognitive components in terms of their changes of the rfMRI metrics across different scales, whereas hand, auditory, mouth, autonomic, reward, basal and default components remain relatively stable over one month. Mapping intra-individual variability of various functional metrics derived from rfMRI voxel-wise further offered a high-resolution landscape of temporal dynamics in functional connectomes. This presents a resource for understanding how individual stable structural connectomes generate their vast functional repertoire [79, 80] and related dynamics [81–83] in enabling action, perception and cognition [73], as well as their alterations, under disease conditions [84].

Driving forces behind the inter-individual variability of brain structure and function are related to both genetic and environmental factors [85]. Such variability of the high-order association cortex is less influenced by genetic factors with their neuroanatomical properties during development, preserving room for environmental factors to exert impacts on the functional variability [86]. With rfMRI, recent studies have increasingly shown inter-individual variability in functional connectivity. One consistent finding is that the variability in the heteromodal association cortex was significantly higher than that in unimodal cortex [39, 87]. Inter-individual variability in connectivity was significantly correlated with the degree of evolutionary cortical expansion [39]. Of note, all previous studies employed seed-based correlation or independent component analysis methods to examine connectivity profiles [88], leaving a lack of inter-individual variability in other functional metrics across the cerebral cortex. Our findings filled this void by assessing the inter-individual variability of six other functional metrics. Specifically, at the global brain level, two network centrality metrics are less variable whereas four other local metrics are relatively more variable across participants. The dorsal attention network, ventral attention network and somatomotor network are more stable across participants whereas the limbic network, default network, frontoparietal control network and visual network demonstrate high inter-individual variability of these functional metrics. Intriguingly, hand, auditory, language, mouth, attention and autonomic components showed small inter-individual variability. In contrast, visual, inhibition, default, basal and reward cognitive components are more variable across participants. Together with vertex-wise high-resolution maps of this variability, the present work delineates a more completed picture of inter-individual variability in functional connectomes, indicating the complexity of different aspects of information processing through connectomes and their individual differences reflected in the human brain intrinsic architecture [1, 89].

By employing a set of canonical templates of the large-scale cortical functional networks [60], the dual regression method offers us the opportunity of examining the brain dynamics in functional connectomics across the one month. This depicts a relatively full picture of the functional connectome regarding its temporal variability and individual differences. One interesting observation is that long-distance functional connectivity between networks or interplays/integrations seem more temporally dynamic than their changes across subjects (Fig 6C). In contrast, short-distance connectivity within the networks or functional specialization remained temporally stable than their between-subject variability (Fig 6D). These findings are consistent with the previous reports at scales of years (brain development and aging across the lifespan) [90–96], which recently have been linked to lifespan changes of behaviors across individuals [97].

Intra-individual and inter-individual variability are two important contributing factors to the reliability of metrics in functional connectomics. Applications, especially in clinical diagnosis, favor metrics with high test-retest reliability, which optimizes a trade-off between the two variability components with low intra-individual variability (more stable across different measuring occasions) and high inter-individual variability (more differentiable across participants), and serves as a necessary condition for high validity of a biomarker [17]. Previous studies have demonstrated moderate to high test-retest reliability of common functional connectome metrics [19], although the sample sizes in these test-retest studies were limited. A recent Consortium for Reliability and Reproducibility (CoRR) released more than 5,000 test-retest multi-modal imaging datasets to the connectomics field [47], providing an open resource for large-scale test-retest reliability exploration in functional connectomics. The present datasets are part of the CoRR datasets. All the preprocessed data and CCS scripts in the present work will be made public to the field soon after the final acceptance of the current work via a data-sharing platform in the Institute of Psychology, Chinese Academy of Sciences. These findings represent the first collection of test-retest reliability for multi-scale and multi-metric characterization of functional human connectomes. As demonstrated in a recent study on reliability-based correction for functional connectivity [98], these test-retest reliability maps generated by the current work can serve as the essential resources for attenuation correction for functional connectomics (i.e., these rfMRI-based metrics) [19] and thus are crucial for the field to guide the use and correction of common functional metrics, as well as their explanation, in applications.

Regional differences in signal-to-noise ratio [60, 61] have been reported, and their influences on test-retest reliability need further investigation in future. Methodological issues such as smoothing, filtering and global signal regression could disturb individual variability [99, 100]. Although these confounding variables have been handled to some degree here, more accurate and sophisticated solutions should be developed in future studies [15, 19, 51]. Based upon a more richly-sampled test-retest samples, the current work provided consistent results with those derived from our recent meta-summary on previous test-retest studies [19]. Although small potential differences existed between the two studies, generally speaking, we would recommend the readers to use them as two complementary references for guiding the use of rfMRI-derived metrics for human brain functional connectomics in application. One big improvement we made in the present work is to compute all the metrics on the cortical surfaces and thus leads to increases of the reliability of some metrics (e.g., seed-based functional connectivity [22]).

## Supporting Information

S1 Table Network summary on individual differences and test-retest reliability of common rfMRI metrics at local scales. (PDF)

S2 Table Cognition summary on individual differences and test-retest reliability of common rfMRI metrics at local scales. (PDF)

S3 Table Network summary on individual differences and test-retest reliability of common rfMRI metrics at meso scales. (PDF)

S4 Table Cognition summary on individual differences and test-retest reliability of common rfMRI metrics at meso scales. (PDF)

S5 Table Network summary on individual differences and test-retest reliability of common rfMRI metrics at global scales. (PDF)

S6 Table Cognition summary on individual differences and test-retest reliability of common rfMRI metrics at global scales. (PDF)
