# Supplementary material for: Individual Variability and Test-Retest Reliability Revealed by Ten Repeated Resting-State Brain Scans over One Month
Source: PLoS One. 2015 Dec 29;10(12):e0144963. doi: 10.1371/journal.pone.0144963 (PMC4694646; doi:10.1371/journal.pone.0144963)
Supplement: S1 Table — (PDF) [file pone.0144963.s001.pdf]

Table S1: Network		Visual		SomMot		DorsAttn		VentAttn		Limbic		Control		Default	
		Mean	Perc	Mean	Perc	Mean	Perc	Mean	Perc	Mean	Perc	Mean	Perc	Mean	Perc
ALFF	ICC	67%	16%	59%	22%	68%	13%	63%	14%	60%	1%	68%	14%	66%	21%
	IntraVar	24%	5%	26%	6%	26%	4%	32%	27%	37%	4%	30%	16%	32%	38%
	InterVar	51%	16%	40%	13%	57%	14%	57%	15%	57%	1%	66%	16%	63%	24%
fALFF	ICC	49%	17%	44%	20%	51%	15%	43%	12%	30%	0%	49%	15%	49%	22%
	IntraVar	38%	13%	37%	12%	38%	9%	45%	19%	58%	2%	43%	18%	44%	28%
	InterVar	37%	14%	29%	7%	41%	16%	34%	11%	26%	0%	43%	20%	43%	31%
ReHo1	ICC	64%	16%	57%	22%	63%	13%	62%	14%	53%	1%	66%	14%	64%	21%
	IntraVar	31%	13%	28%	7%	30%	8%	33%	18%	44%	5%	31%	14%	34%	33%
	InterVar	58%	18%	39%	12%	54%	13%	56%	15%	50%	1%	62%	16%	59%	24%
ReHo2	ICC	63%	16%	56%	22%	61%	13%	61%	14%	55%	1%	63%	14%	61%	20%
	IntraVar	31%	11%	24%	4%	30%	8%	32%	18%	42%	4%	33%	17%	35%	40%
	InterVar	54%	19%	32%	8%	48%	13%	51%	15%	50%	1%	57%	18%	55%	26%
