# Supplementary material for: Individual Variability and Test-Retest Reliability Revealed by Ten Repeated Resting-State Brain Scans over One Month
Source: PLoS One. 2015 Dec 29;10(12):e0144963. doi: 10.1371/journal.pone.0144963 (PMC4694646; doi:10.1371/journal.pone.0144963)
Supplement: S2 Table — (PDF) [file pone.0144963.s002.pdf]

Table S2: Cognition		Hand	Mouth	Auditory	Visual	Language	Attention	Autonomic	Inhibition	Working Memory	Default	Basal	Reward
ALFF	ICC	0.62	0.63	0.64	0.66	0.65	0.67	0.62	0.64	0.67	0.66	0.64	0.62
	IntraVar	0.27	0.28	0.30	0.26	0.30	0.26	0.31	0.31	0.29	0.29	0.31	0.33
	InterVar	0.47	0.48	0.54	0.53	0.56	0.56	0.53	0.58	0.61	0.58	0.56	0.54
fALFF	ICC	0.46	0.45	0.47	0.48	0.47	0.49	0.43	0.45	0.49	0.48	0.45	0.43
	IntraVar	0.38	0.40	0.40	0.39	0.42	0.39	0.43	0.44	0.42	0.41	0.44	0.45
	InterVar	0.33	0.34	0.36	0.37	0.38	0.39	0.33	0.37	0.40	0.40	0.36	0.35
ReHo1	ICC	0.60	0.61	0.63	0.64	0.65	0.63	0.61	0.64	0.65	0.64	0.62	0.61
	IntraVar	0.29	0.31	0.31	0.31	0.31	0.30	0.32	0.32	0.30	0.32	0.34	0.34
	InterVar	0.46	0.50	0.54	0.56	0.58	0.54	0.52	0.57	0.59	0.57	0.55	0.54
ReHo2	ICC	0.59	0.59	0.62	0.62	0.62	0.61	0.59	0.61	0.63	0.62	0.60	0.59
	IntraVar	0.27	0.30	0.29	0.31	0.31	0.30	0.31	0.32	0.31	0.32	0.34	0.34
	InterVar	0.40	0.44	0.49	0.51	0.53	0.49	0.46	0.52	0.54	0.53	0.51	0.49
