# Supplementary material for: Individual Variability and Test-Retest Reliability Revealed by Ten Repeated Resting-State Brain Scans over One Month
Source: PLoS One. 2015 Dec 29;10(12):e0144963. doi: 10.1371/journal.pone.0144963 (PMC4694646; doi:10.1371/journal.pone.0144963)
Supplement: S3 Table — (PDF) [file pone.0144963.s003.pdf]

Table S3: Network		Visual		SomMot		DorsAttn		VentAttn		Limbic		Control		Default	
		Mean	Perc	Mean	Perc	Mean	Perc	Mean	Perc	Mean	Perc	Mean	Perc	Mean	Perc
SFC	ICC	33%	8%	29%	5%	40%	11%	41%	13%	41%	1%	56%	24%	58%	37%
	IntraVar	47%	30%	36%	12%	38%	13%	36%	10%	51%	3%	37%	12%	38%	21%
	InterVar	24%	5%	16%	1%	28%	5%	28%	8%	35%	1%	48%	29%	54%	50%
DR-Visual	ICC	61%	52%	25%	8%	46%	26%	29%	7%	15%	0%	26%	4%	24%	4%
	IntraVar	39%	8%	75%	26%	53%	10%	70%	16%	82%	1%	72%	15%	74%	23%
	InterVar	60%	51%	25%	9%	46%	25%	29%	7%	14%	0%	26%	4%	23%	4%
DR-SomMot	ICC	31%	7%	43%	36%	43%	19%	40%	17%	19%	0%	34%	10%	31%	10%
	IntraVar	67%	16%	55%	22%	56%	12%	59%	13%	80%	1%	64%	14%	67%	21%
	InterVar	30%	7%	43%	35%	43%	19%	40%	17%	19%	0%	34%	11%	30%	10%
DR-DorsAttn	ICC	36%	13%	35%	18%	63%	29%	38%	15%	26%	0%	42%	16%	30%	9%
	IntraVar	62%	17%	64%	26%	35%	4%	61%	15%	71%	1%	57%	13%	68%	24%
	InterVar	35%	13%	34%	18%	62%	29%	38%	15%	25%	0%	42%	16%	30%	9%
DR-VentAttn	ICC	32%	5%	42%	18%	55%	16%	61%	19%	31%	1%	60%	18%	50%	23%
	IntraVar	65%	22%	58%	29%	44%	11%	38%	8%	67%	2%	39%	8%	48%	21%
	InterVar	31%	4%	42%	18%	54%	16%	60%	19%	31%	0%	60%	18%	50%	23%
DR-Limbic	ICC	19%	4%	17%	6%	22%	6%	24%	13%	45%	9%	30%	25%	30%	36%
	IntraVar	79%	16%	83%	23%	77%	13%	75%	14%	52%	1%	68%	14%	68%	21%
	InterVar	19%	4%	17%	7%	22%	7%	24%	14%	43%	8%	30%	26%	29%	35%
DR-Control	ICC	30%	6%	24%	4%	66%	20%	46%	14%	33%	1%	71%	23%	62%	32%
	IntraVar	68%	23%	75%	35%	33%	7%	53%	16%	66%	2%	29%	4%	37%	12%
	InterVar	29%	6%	23%	4%	67%	20%	46%	14%	32%	1%	72%	23%	63%	33%
DR-Default	ICC	31%	6%	31%	8%	49%	15%	46%	15%	41%	1%	64%	22%	65%	34%
	IntraVar	68%	22%	68%	31%	51%	14%	54%	16%	57%	2%	35%	6%	35%	9%
	InterVar	31%	6%	31%	8%	49%	15%	46%	15%	40%	1%	65%	22%	65%	33%
