# Supplementary material for: Individual Variability and Test-Retest Reliability Revealed by Ten Repeated Resting-State Brain Scans over One Month
Source: PLoS One. 2015 Dec 29;10(12):e0144963. doi: 10.1371/journal.pone.0144963 (PMC4694646; doi:10.1371/journal.pone.0144963)
Supplement: S4 Table — (PDF) [file pone.0144963.s004.pdf]

Table S4: Cognition		Hand	Mouth	Auditory	Visual	Language	Attention	Autonomic	Inhibition	Working Memory	Default	Basal	Reward
SFC	ICC	0.34	0.35	0.44	0.36	0.44	0.39	0.38	0.48	0.47	0.50	0.47	0.44
	IntraVar	0.37	0.39	0.37	0.44	0.39	0.39	0.37	0.37	0.39	0.38	0.39	0.41
	InterVar	0.21	0.23	0.33	0.26	0.33	0.29	0.26	0.38	0.37	0.42	0.39	0.36
DR-Visual	ICC	0.30	0.30	0.26	0.51	0.35	0.43	0.27	0.29	0.35	0.33	0.33	0.30
	IntraVar	0.70	0.70	0.73	0.49	0.65	0.56	0.73	0.70	0.64	0.65	0.66	0.69
	InterVar	0.30	0.30	0.25	0.51	0.35	0.43	0.27	0.29	0.34	0.33	0.32	0.30
DR-SomMot	ICC	0.43	0.42	0.38	0.36	0.35	0.39	0.39	0.36	0.37	0.34	0.32	0.32
	IntraVar	0.56	0.57	0.61	0.63	0.65	0.59	0.60	0.63	0.62	0.64	0.66	0.67
	InterVar	0.43	0.42	0.37	0.35	0.35	0.39	0.38	0.36	0.37	0.33	0.32	0.31
DR-DorsAttn	ICC	0.44	0.37	0.37	0.47	0.40	0.51	0.41	0.36	0.51	0.36	0.34	0.33
	IntraVar	0.55	0.62	0.61	0.52	0.59	0.48	0.59	0.62	0.49	0.62	0.64	0.66
	InterVar	0.43	0.36	0.37	0.46	0.40	0.50	0.40	0.36	0.51	0.36	0.33	0.33
DR-VentAttn	ICC	0.49	0.49	0.50	0.43	0.52	0.53	0.52	0.59	0.58	0.47	0.47	0.48
	IntraVar	0.51	0.50	0.49	0.56	0.47	0.46	0.46	0.40	0.42	0.51	0.52	0.50
	InterVar	0.48	0.49	0.50	0.42	0.52	0.52	0.52	0.59	0.58	0.46	0.46	0.48
DR-Limbic	ICC	0.18	0.18	0.27	0.21	0.27	0.20	0.23	0.27	0.26	0.26	0.28	0.26
	IntraVar	0.82	0.81	0.72	0.78	0.73	0.78	0.76	0.72	0.73	0.72	0.70	0.73
	InterVar	0.18	0.18	0.27	0.21	0.27	0.20	0.23	0.27	0.26	0.26	0.28	0.26
DR-Control	ICC	0.40	0.35	0.43	0.47	0.59	0.57	0.42	0.61	0.70	0.50	0.50	0.51
	IntraVar	0.59	0.64	0.55	0.52	0.41	0.42	0.57	0.39	0.30	0.49	0.49	0.48
	InterVar	0.40	0.35	0.43	0.48	0.59	0.58	0.42	0.61	0.71	0.50	0.50	0.51
DR-Default	ICC	0.39	0.37	0.50	0.40	0.52	0.46	0.42	0.56	0.57	0.53	0.51	0.49
	IntraVar	0.61	0.62	0.50	0.60	0.48	0.53	0.58	0.44	0.43	0.46	0.49	0.51
	InterVar	0.39	0.37	0.50	0.40	0.52	0.46	0.42	0.56	0.57	0.53	0.51	0.49
