# Supplementary material for: Individual Variability and Test-Retest Reliability Revealed by Ten Repeated Resting-State Brain Scans over One Month
Source: PLoS One. 2015 Dec 29;10(12):e0144963. doi: 10.1371/journal.pone.0144963 (PMC4694646; doi:10.1371/journal.pone.0144963)
Supplement: S5 Table — (PDF) [file pone.0144963.s005.pdf]

Table S5: Network		Visual		SomMot		DorsAttn		VentAttn		Limbic		Control		Default	
		Mean	Perc	Mean	Perc	Mean	Perc	Mean	Perc	Mean	Perc	Mean	Perc	Mean	Perc
DCw	ICC	40%	21%	39%	28%	32%	6%	40%	17%	33%	1%	33%	8%	37%	19%
	IntraVar	58%	17%	46%	16%	64%	14%	59%	15%	66%	1%	63%	15%	61%	22%
	InterVar	40%	26%	30%	12%	31%	7%	39%	21%	33%	1%	31%	9%	36%	24%
ECw	ICC	30%	20%	34%	61%	21%	5%	26%	7%	19%	0%	14%	2%	18%	4%
	IntraVar	68%	16%	64%	22%	77%	13%	70%	14%	79%	1%	80%	14%	79%	21%
	InterVar	30%	24%	33%	57%	20%	5%	25%	6%	18%	1%	13%	2%	18%	5%
