# Supplementary material for: Individual Variability and Test-Retest Reliability Revealed by Ten Repeated Resting-State Brain Scans over One Month
Source: PLoS One. 2015 Dec 29;10(12):e0144963. doi: 10.1371/journal.pone.0144963 (PMC4694646; doi:10.1371/journal.pone.0144963)
Supplement: S6 Table — (PDF) [file pone.0144963.s006.pdf]

| Table S6: Cognition | | Hand | Mouth | Auditory | Visual | Language | Attention | Autonomic | Inhibition | Working Memory | Default | Basal | Reward |
|---|---|---|---|---|---|---|---|---|---|---|---|---|---|
| DCw | ICC | 0.38 | 0.37 | 0.39 | 0.38 | 0.35 | 0.35 | 0.38 | 0.37 | 0.34 | 0.37 | 0.37 | 0.36 |
| | IntraVar | 0.53 | 0.56 | 0.57 | 0.60 | 0.62 | 0.61 | 0.56 | 0.61 | 0.62 | 0.60 | 0.61 | 0.61 |
| | InterVar | 0.32 | 0.33 | 0.36 | 0.36 | 0.34 | 0.33 | 0.35 | 0.35 | 0.33 | 0.35 | 0.35 | 0.35 |
| ECw | ICC | 0.28 | 0.26 | 0.25 | 0.27 | 0.21 | 0.23 | 0.25 | 0.22 | 0.18 | 0.22 | 0.22 | 0.22 |
| | IntraVar | 0.70 | 0.71 | 0.72 | 0.72 | 0.76 | 0.74 | 0.71 | 0.74 | 0.77 | 0.75 | 0.75 | 0.74 |
| | InterVar | 0.27 | 0.25 | 0.25 | 0.26 | 0.20 | 0.22 | 0.24 | 0.21 | 0.18 | 0.21 | 0.22 | 0.21 |
